# 厄洛替尼致间质性肺病4例并文献复习

**DOI:** 10.3779/j.issn.1009-3419.2012.08.08

**Published:** 2012-08-20

**Authors:** 小玲 吴, 广辉 高, 胜祥 任, 彩存 周

**Affiliations:** 1 643000 自贡，自贡市第一人民医院呼吸科 Department of Respiratory Disease, Zigong First Pepole's Hospital, Zigong 643000, China; 2 200433 上海，同济大学附属上海市肺科医院肿瘤科 Department of Oncology, Tongji University Affiliated Shanghai Pulmonary Hospital, Shanghai 200433, China

**Keywords:** 肺肿瘤, 厄洛替尼, 表皮生长因子受体, Lung neoplasms, Erlotinib, Epidermal growth factor receptor

## Abstract

厄洛替尼是一种口服表皮生长因子受体酪氨酸激酶抑制剂（epidermal growth factor receptor tyrosine kinase inhibitor, EGFR-TKI），广泛应用于晚期非小细胞肺癌的治疗。尽管目前认为这类药物安全性良好，但可诱发严重的间质性肺病（interstitial lung disease, ILD）。文献中对吉非替尼所致ILD的报道较多，而对厄洛替尼所致的ILD报道较少。现报道上海市肺科医院4例厄洛替尼所致ILD，并复习相关文献，以使临床医生提高警惕。监测正在服用厄洛替尼患者的肺部症状、体征，做到及时诊断、尽早治疗对于厄洛替尼所致ILD的预后尤为重要。

## Abstract (en)

Erlotinib is an agent of oral epidermal growth factor receptor (EGFR) tyrosine kinase inhibitors which are used for non-small cell lung cancer. Although this class of agents is considered to be relatively safe, the most serious, but rare, adverse reaction is drug-associated interstitial lung disease (ILD). ILD induced by gefitinib been often described, but the ILD induced by erlotinib is relatively less well known. We here describle four cases of ILD related to erlotinib and review recent literatures to help physicians earlier alert erlotinib-induced ILD. It is important to carefully monitor pulmonary symptoms in all patients who are receiving erlotinib. Early diagnosis and timely intervention is critical in the treatment of drug-induced ILD.

## 1 临床资料

病例1：男，47岁，2007年12月20日因咳嗽、咯痰1个月入院。既往无肺部疾病及结缔组织疾病史。体检无阳性体征。气管镜检查示气管及各级支气管未见明显异常。胸部CT示右肺中叶软组织密度影。骨扫描示右后第8肋异常浓聚。头颅MRI示多发缺血灶。2007年12月31日在全麻下行右肺中叶切除术。术后病理显示右肺中叶中分化腺癌，累及脏层胸膜，切缘（-），送检淋巴结第4组、7组（+），第3组、9组、10组、11组均（-）。表皮生长因子受体（epidermal growth factor receptor, EGFR）基因突变检测阳性。患者术后1个月予长春瑞滨+顺铂方案化疗4周期后随访。2009年7月25日胸部CT提示疾病进展，于2009年8月1日开始口服厄洛替尼治疗（150 mg/d），服药24天后出现发热（最高40 ℃）、干咳、呼吸困难，并逐渐加重。查体：双下肺闻及湿啰音。胸部CT：右肺中叶切除术后，双肺散在斑片状毛玻璃影及条索影，双侧少量胸腔积液。血常规示白细胞12.4×10^9^/L，中性粒细胞比例71.3%，胸水提示渗出液细菌及真菌培养（-），血气分析示低氧血症，血沉、C反应蛋白、肝功、肾功、电解质正常，呼吸道病原体检测、痰细菌、真菌及结核菌培养均阴性。诊断考虑为间质性肺病（interstitial lung disease, ILD），停用厄洛替尼，予头孢哌酮/他唑巴坦预防性抗感染、甲基强的松龙（40 mg/d，静滴）抗炎等对症治疗。1周后咳嗽、呼吸困难症状缓解，复查CT提示肺内急性渗出病灶完全吸收，停用抗生素，口服强的松并逐渐减量（起始40 mg/d，以后每周减量5 mg/d直至停药）（图 1）。

**1:**
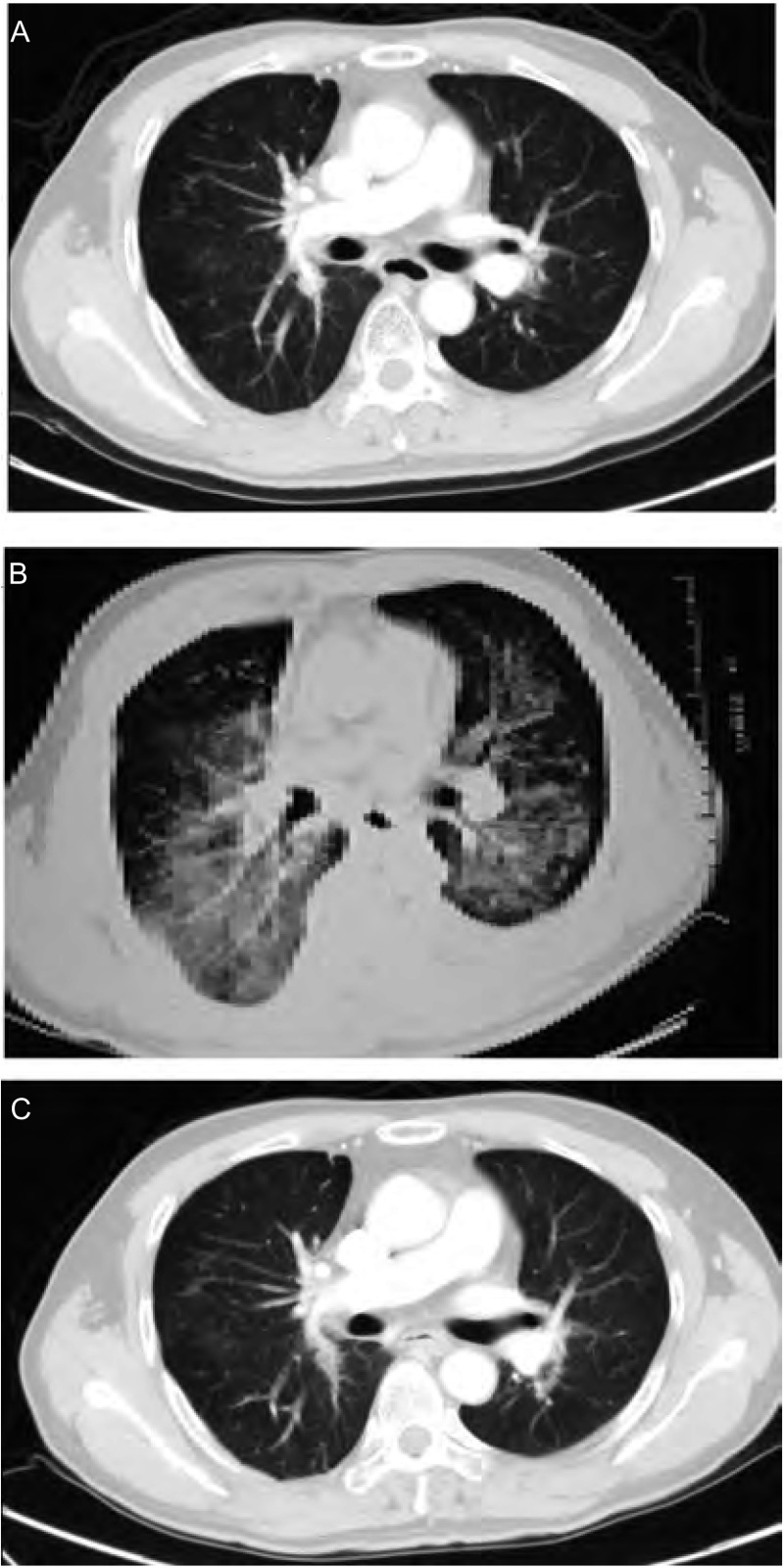
病例1厄洛替尼治疗前后及糖皮质激素治疗后胸部CT变化 Chest CT scans before and after erlotinib treatment and after corticosteroid treatment. A: Chest CT scan before erlotinib treatment; B: Chest CT scan after 24 days of erlotinib treatment; C: Chest CT scan after 7 days of corticosteroid treatment.

病例2：女，76岁，既往无肺部疾病及结缔组织疾病史。2008年12月14日外院诊断为右肺腺癌伴双肺转移T4N2M1a-Ⅳ期（两肺），2008年12月19日开始口服厄洛替尼（150 mg/d）治疗，1个月后复查CT提示原发病灶缩小，服药70天后出现发热伴咳嗽、胸闷，多为干咳、偶有少量白色粘液痰。查体：双肺闻及细湿啰音。血常规示白细胞17.6×10^9^/L，中性粒细胞比例81.25%，血气分析示低氧血症，胸部CT示右肺上叶肺癌伴双肺转移、双肺上叶为主多发片状磨玻璃影（与之前CT比较为新出现）、双侧少量胸腔积液（为新出现）、纵隔淋巴结肿大，痰细菌、真菌及结核菌培养均阴性。诊断考虑ILD，停用厄洛替尼并予头孢西丁预防性抗感染、甲基强的松龙（40 mg/d，静滴）抗炎治疗1周，继之口服强的松并逐渐减量（起始30 mg/d，以后每周减量5 mg/d直至停药），症状明显好转。10天后复查胸部CT示右肺上叶肺癌伴双肺转移、双肺多发片状磨玻璃影较前好转。见图 2。

**2:**
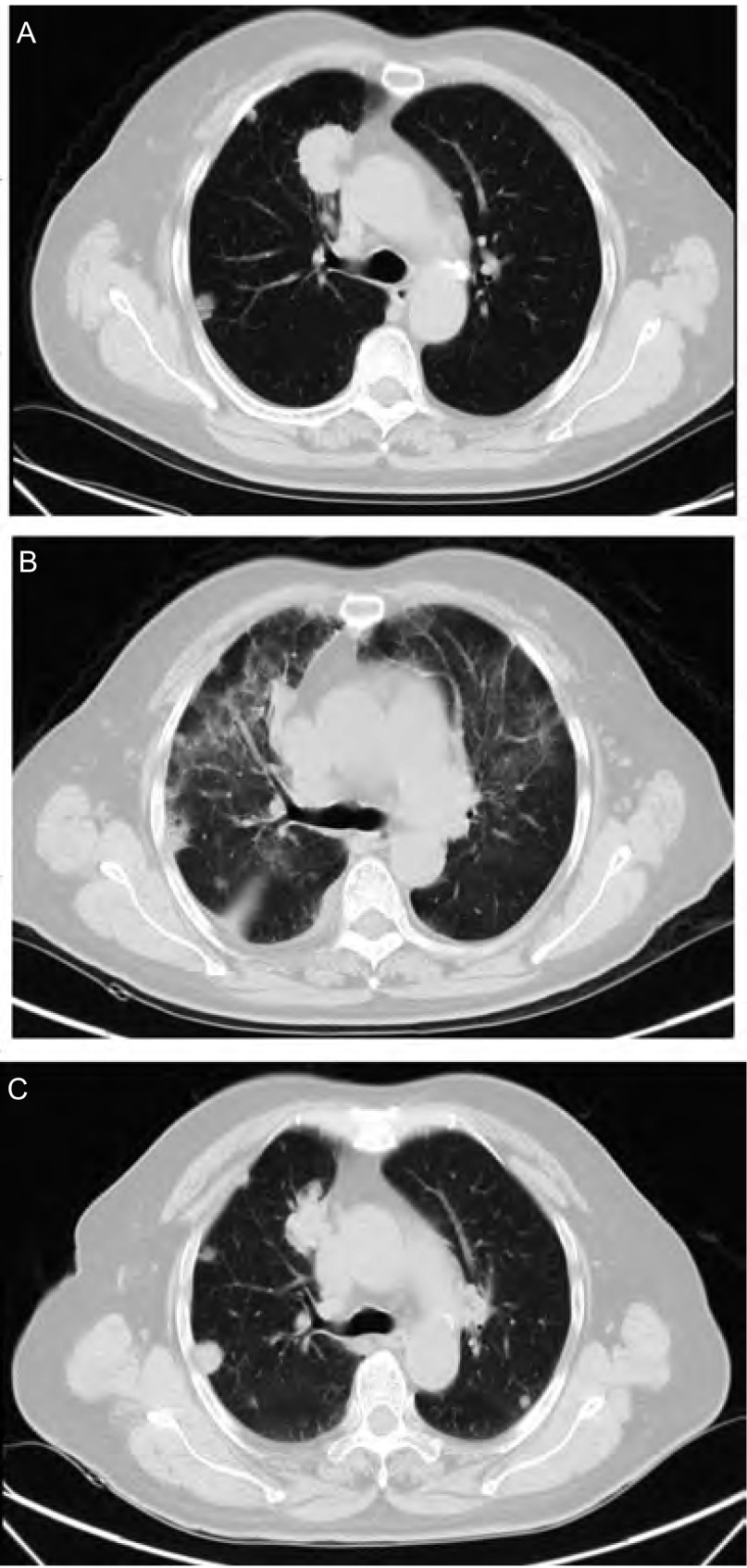
病例2厄洛替尼治疗前后及糖皮质激素治疗后胸部CT变化 Chest CT scans before and after erlotinib treatment and after corticosteroid treatment. A: Chest CT scan before erlotinib treatment; B: Chest CT scan after 70 days of erlotinib treatment; C: Chest CT scan after 10 days of corticosteroid treatment.

病例3：男，58岁，2011年12月22日因咳嗽、胸闷入院。既往无肺部疾病及结缔组织疾病史。外院胸部CT示双肺广泛分布粟粒、斑点、类结节密度增高影，纵隔淋巴结肿大及左侧胸腔积液。经气管镜刷检及活检病理提示为左下肺腺癌。*EGFR*基因突变检测为阳性。诊断为左肺腺癌T4N2M1a-Ⅳ期（两肺）。2011年12月31日参加临床研究开始口服厄洛替尼（150 mg/d）治疗。服药21天后出现干嗽、呼吸困难。查体：双肺满布细湿啰音，左肺闻及干鸣音，胸部CT提示双肺散在结节影、条索影、网格影及实变影，左肺实变较前增多。血常规示白细胞6.3×10^9^/L，中性粒细胞比例84.3%，血气分析（鼻导管吸氧3 L/min）示低氧血症，痰细菌、真菌培养均阴性。诊断考虑为ILD，停用厄洛替尼，予头孢西丁预防性抗感染、甲基强的松龙（40 mg/d，静滴）抗炎及对症支持治疗，继之口服强的松起始30 mg/d，1周后减量5 mg/d。2周后症状缓解出院，继续口服强的松（每周减量5 mg/d直至停药）。3周后随访无咳嗽、胸闷主诉，查体双下肺闻及Velcro啰音，复查CT示双肺散在结节影、条索影、网格影及实变影，双肺病变较前好转。见图 3。

**3:**
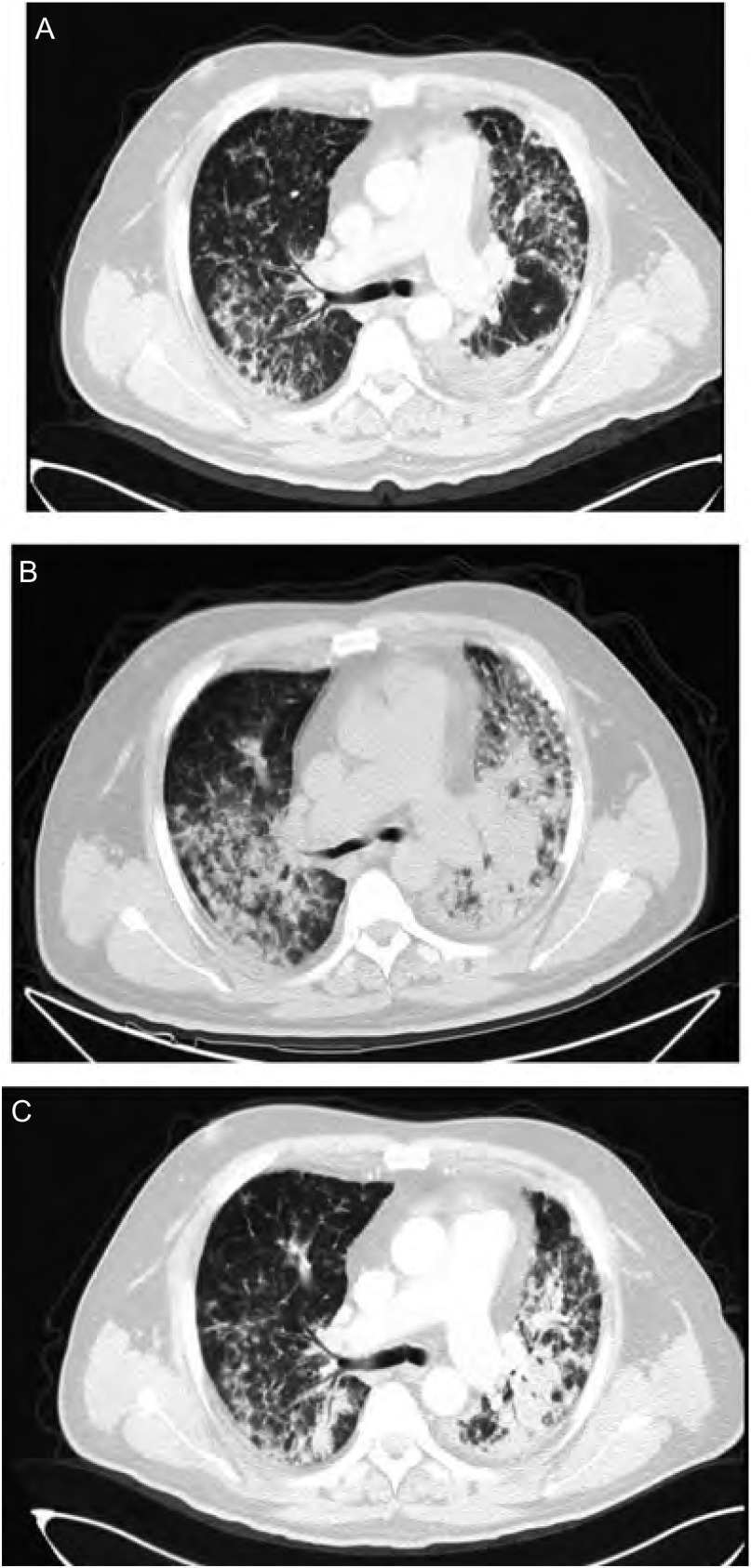
病例3厄洛替尼治疗前后及糖皮质激素治疗后胸部CT变化 Chest CT scans before and after erlotinib treatment and after corticosteroid treatment. A: Chest CT scan before erlotinib treatment; B: Chest CT scan after 21 days of erlotinib treatment; C: Chest CT scan after 21 days of corticosteroid treatment.

病例4：男，65岁，既往无肺部疾病及结缔组织疾病史。2003年12月19日确诊为右肺上叶腺癌，行右肺上叶切除术，术后行4周期紫杉醇+卡铂辅助化疗。2007年8月28日随访发现右肺下叶复发，再次行右全肺切除术，术后予多西他赛+卡铂方案化疗4周期。2011年11月14日随访发现左肺转移伴多发骨转移，予培美曲塞+顺铂方案化疗4周期，于2012年2月29日予厄洛替尼（150 mg/d）维持治疗。服药22天后出现胸闷、气促伴发热。查体：右肺无呼吸音，左肺呼吸音粗。胸部CT提示右肺切除术后左肺多发转移瘤伴斑片影、实变影。血常规示白细胞9.0×10^9^/L，中性粒细胞比例78.9%，血气分析（鼻导管吸氧3 L/min）示低氧血症，痰细菌、真菌培养均阴性。诊断考虑为ILD，停用厄洛替尼，头孢西丁预防性抗感染、甲基强的松龙（40 mg/d，静滴）抗炎及对症支持治疗，继之口服强的松（起始30 mg/d，1周后每周减量5 mg/d直至停药）。10天后症状缓解，复查CT示左肺斑片灶较前吸收。见图 4。

**4:**
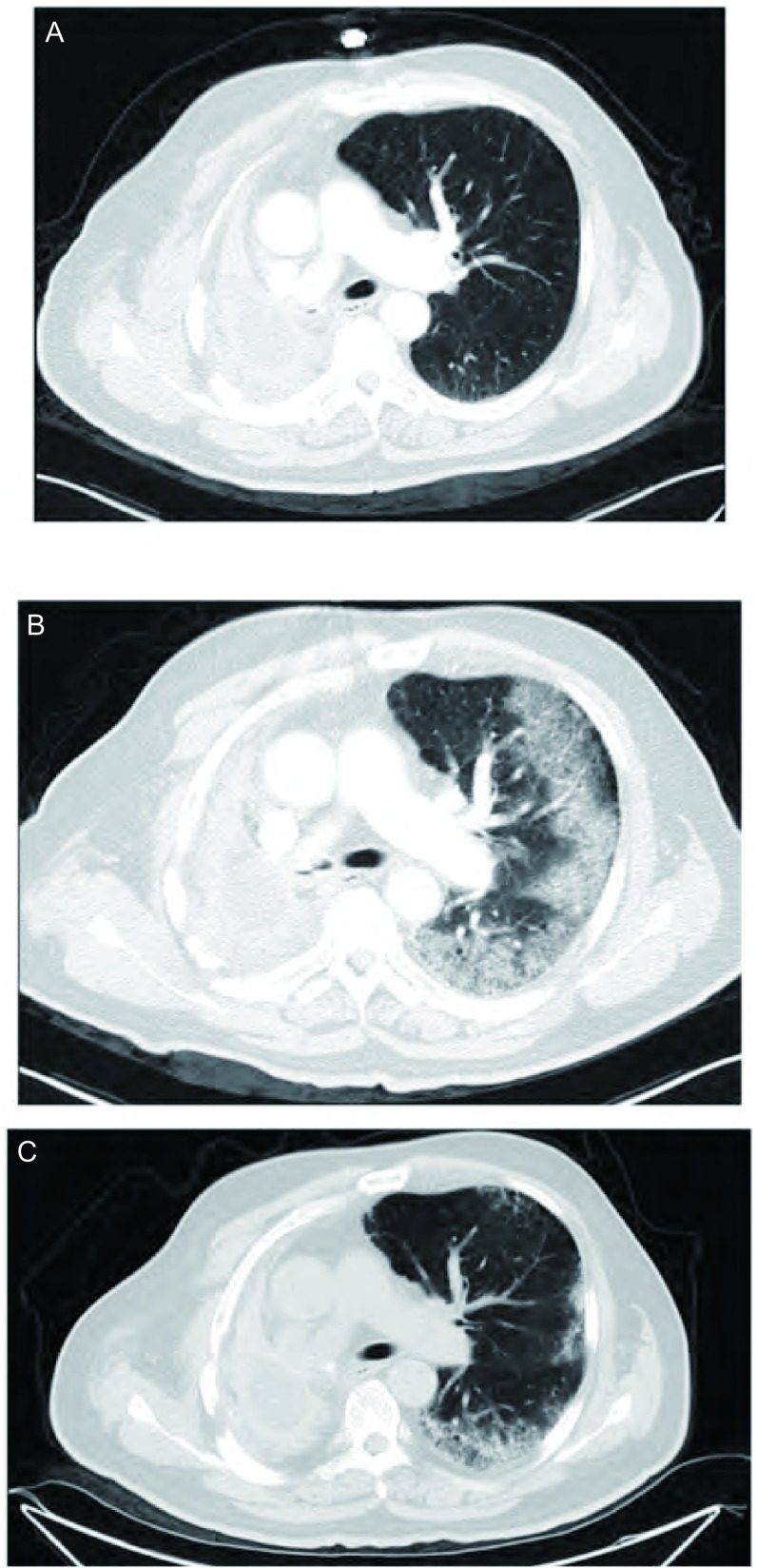
病例4厄洛替尼治疗前后及糖皮质激素治疗后胸部CT变化 Chest CT scans before and after erlotinib treatment and after corticosteroid treatment. A: Chest CT scan before erlotinib treatment; B: Chest CT scan after 22 days of erlotinib treatment; C: Chest CT scan after 10 days of corticosteroid treatment.

## 2 讨论

吉非替尼和厄洛替尼均为小分子表皮生长因子受体酪氨酸激酶抑制剂（epidermal growth factor receptor tyrosine kinase inhibitor, EGFR-TKI），选择性地作用于EGFR的酪氨酸激酶，阻止其下游的信号传导，从而抑制肿瘤血管生成、增殖、促进细胞凋亡。EGFR-TKI现已广泛用于有*EGFR*基因突变的肺腺癌的一线治疗，也用于晚期非小细胞肺癌的二线、三线及维持治疗，尤其对亚裔女性、非吸烟、腺癌患者效果较好^[1, 2]^，常见的不良反应有皮肤反应（皮疹、痤疮、皮肤干燥等）、消化道反应（腹泻、恶心和呕吐），大多表现为轻中度、不需停药，而发生率低、死亡率高的副反应为间质性肺病。本文报道的4例厄洛替尼导致的ILD，由于早期诊断、及时停药并积极治疗，预后较好。

TKI致ILD发生率约为1%，文献中对吉非替尼所致ILD的报道较多，日本报道吉非替尼发生ILD几率为3.5%，死亡率为1.6%^[3]^，美国报道厄洛替尼肺损伤发病率为0.8%，发病时间从厄洛替尼治疗后5天至9月不等，中位时间为39天^[4]^，部分可因呼吸衰竭死亡，国内尚无确切发生率的统计报道。

TKI导致ILD的机制尚不明确。已知EGFR参与肺部损伤的修复，推测EGFR-TKI通过抑制EGFR表达、减少组织中EGFR含量、降低EGFR的活性、抑制气道上皮细胞的生长和修复、妨碍肺部损伤的修复从而导致ILD。另有研究^[5]^发现吉非替尼导致间质性肺病患者支气管肺泡灌洗液中干扰素诱导蛋白10的水平增加，提示炎症反应可能参与了TKI诱导ILD的发生。也有推测ILD的发生可能与药物过敏、肿瘤坏死释放出大量的肿瘤坏死因子以及药物加重放射性肺炎等有关。Ⅰ期研究^[6]^显示厄洛替尼（150 mg/d）稳态血药浓度为1.37 μg/mL-1.64 μg/mL，但Tsubata等^[7]^发现厄洛替尼致ILD患者在口服厄洛替尼（150 mg/d）后第6天血药浓度高达3.62 μg/mL，ter Heine^[8]^等也有类似报道。由此推测厄洛替尼引起的肺病可能与药物的血药浓度过高有关。尽管ILD发生机制不明，但多因素分析显示男性、吸烟、合并存在间质性肺炎、既往有肺纤维化、化疗史和一般情况差是高危因素^[3]^。

TKI致ILD目前尚缺乏统一的诊断和治疗标准，主要依据患者服药史、症状、体征及相关辅助检查和肺部影像改变进行诊断，若条件许可取得病理诊断可确定间质性肺疾病的诊断。但大多数患者由于病情进展迅速、病情危重、不能耐受支气管肺泡灌洗及肺穿活检等检查，缺乏排它性确诊依据，给临床诊断和治疗带来一定的困难。目前文献^[9]^报道仅有1例尸检通过组织学证实厄洛替尼治疗2个月后发生的间质性肺疾病导致死亡。

ILD的主要表现为干咳、不同程度的呼吸困难；肺功能检查为限制性通气功能障碍及弥散功能减低，伴低氧血症；影像学上可有多种表现：磨玻璃样改变、多灶性肺实变、片状或弥漫性分布的磨玻璃样阴影、肺实变伴牵拉性支气管扩张，其中表现为弥漫性肺部病变者死亡率最高^[10]^。肿瘤患者病情突然加重，需考虑心功能衰竭引起的急性肺水肿、合并肺部感染、肿瘤进展、合并间质性肺炎，还需与肺泡蛋白沉着症、变应性支气管肺曲菌病、肺结缔组织疾病等进行鉴别。

本文4例患者既往无心脏病史，除心律快外无其它阳性心脏体征，心电图未提示心律不齐及心肌缺血等病变，心肌酶谱、B型脑钠肽正常排除心衰；有咳嗽症状，部分伴发热，双肺闻及湿啰音，白细胞轻度升高，但常规抗感染治疗效果欠佳，而激素治疗效果好；入院时CT均未提示肿瘤进展。4例患者均有呼吸困难、咳嗽，3例伴发热，查体双肺闻及细湿啰音，血气分析提示低氧血症，胸部CT提示双肺弥漫性肺部斑片渗出、磨玻璃影，经激素治疗后好转，结合用药史应考虑ILD可能，而患者既往无慢性肺疾病史，近期无静脉化疗用药，考虑为TKI导致ILD。

ILD治疗方案有单纯激素治疗、激素与免疫抑制剂联合治疗，目前以糖皮质激素为主，但尚无统一治疗方案。TKI导致的ILD病情发展迅速，即使给予大剂量激素，病情仍可能加重，死亡率高。但本文4例患者在服药后出现相关症状，建立临床诊断后立即停用厄洛替尼，根据病情予糖皮质激素、预防感染及对症支持治疗，预后较好，无1例死亡，此后随访也未再出现ILD，这与对该病的重视、及早发现和积极治疗有关。

对于TKI导致的ILD，除了需要考虑疾病本身的治疗外，肿瘤下一步的治疗策略也是需要进一步探讨的问题。因考虑再用或换用TKI可能再次发生ILD，目前大多数患者转为合适的化疗方案。本文4例患者也在ILD缓解后根据病情及既往治疗情况选用化疗，除病例2患者1年后因肿瘤进展死亡外，其余3例均随访至2012年4月且疾病稳定。但目前使用TKI的患者多为晚期非小细胞肺癌，且已多次化疗失败或为一般情况较差而不能耐受化疗者。Takamochi等^[11]^报道了1例口服吉非替尼诱发ILD后因肿瘤进展、一般情况差无法耐受化疗，减量应用吉非替尼来延缓疾病进展，也有报道^[12, 13]^对服用吉非替尼后发生ILD的患者换用厄洛替尼延缓疾病进展。

尽管TKI致ILD发病率不高，但大多患者既往曾接受化疗、放疗，且多为晚期非小细胞肺癌患者，机体免疫力低下，一旦发病则病情危重，死亡率极高，故临床医生应对TKI致ILD提高警惕，做到早诊断、早停药、早治疗，从而提高患者的生存率，而该类患者的后续治疗有待于更多的观察进一步明确。

## References

[1] Zhou C, Wu YL, Chen G (2011). Erlotinib versus chemotherapy as first-line treatment for patients with advanced *EGFR* mutation-positive non-small-cell lung cancer (OPTIMAL, CTONG-0802): a multicentre, open-label, randomised, phase 3 study. Lancet Oncol.

[2] Cappuzzo F, Ciuleanu T, Stelmakh L (2010). Erlotinib as maintenance treatment in advanced non-small-lung cancer: a multi-centre, randomised, placebo-controlled phase 3 study. Lancet Oncol.

[3] Takano T, Ohe Y, Kusumoto M (2004). Risk factors for interstitial lung disease and predictive factors for tumor response in patients with advanced non-small cell lung cancer treated with gefitinib. Lung Cancer.

[4] Cohen MH, Johnson JR, Chen YF (2005). FDA approval summary: erlotinib (tarceva) tablets. Oncologists.

[5] Kataoka K, Taniguchi H, Hasegawa Y (2006). Interstitial lung disease associated with gefitinib. Respir Med.

[6] Yamamoto N, Horiike A, Fujisaka Y (2008). Phase Ⅰ dose-finding and pharmacokinetic study of the oral epidermal growth factor receptor tyrosine kinase inhibitor Ro50-8231 (erlotinib) in Japanese patients with solid tumors. Cancer Chemother Pharmacol.

[7] Tsubata Y, Hamada A, Sutani A (2012). Erlotinib-induced acute interstitial lung disease associated with extreme elevation of the plasma concentration in an elderly non-small-cell lung cancer patient. J Cancer Res Ther.

[8] ter Heine R, van den Bosch RT, Schaefer-Prokop CM (2012). Fatal interstitial lung disease associated with high erlotinib and metabolite levels. A case report and a review of the literature. Lung Cancer.

[9] Makris D, Scherpereel A, Copin MC (2007). Fatal interstitial lung disease associated with oral erlotinib therapy for lung cancer. BMC Cancer.

[10] Endo M, Johkoh T, Kimura K (2006). Imaging of gefitinib-related interstitial lung disease: multi-institutional analysis by the West Japan Thoracic Oncology Group. Lung Cancer.

[11] Takamochi K, Suzuki K, Bashar AH (2007). Readministration of gefitinib in a responder after treatment discontinuation due to gefinitib-related interstitial lung disease: a case report. J Med Case Rep.

[12] Tian Q, Chen LA (2011). Erlotinib achieved partial response in a non-small cell lung cancer patient with gefitinib-induced interstitial lung disease. Case Rep Oncol.

[13] Fukui T, Otani S, Hataishi R (2010). Successful rechallenge with erlotinib in a patient with *EGFR*-mutant lung adenocarcinoma who developed gefitinib-related interstitial lung disease. Cancer Chemother Pharmacol.

